# Long-Term Effects of Acute Stress on the Prefrontal-Limbic System in the Healthy Adult

**DOI:** 10.1371/journal.pone.0168315

**Published:** 2017-01-03

**Authors:** Yu Li, Xin Hou, Dongtao Wei, Xue Du, Qinglin Zhang, Guangyuan Liu, Jiang Qiu

**Affiliations:** 1 Key Laboratory of Cognition and Personality (SWU), Ministry of Education, Chongqing, China; 2 School of Psychology, Southwest University, Chongqing, China; 3 College of Electronic and Information Engineering, Chongqing, China; University of Pennsylvania Perelman School of Medicine, UNITED STATES

## Abstract

Most people are exposed to at least one traumatic event during the course of their lives, but large numbers of people do not develop posttraumatic stress disorders. Although previous studies have shown that repeated and chronic stress change the brain’s structure and function, few studies have focused on the long-term effects of acute stressful exposure in a nonclinical sample, especially the morphology and functional connectivity changes in brain regions implicated in emotional reactivity and emotion regulation. Forty-one months after the 5/12 Wenchuan earthquake, we investigated the effects of trauma exposure on the structure and functional connectivity of the brains of trauma-exposed healthy individuals compared with healthy controls matched for age, sex, and education. We then used machine-learning algorithms with the brain structural features to distinguish between the two groups at an individual level. In the trauma-exposed healthy individuals, our results showed greater gray matter density in prefrontal-limbic brain systems, including the dorsal anterior cingulate cortex, medial prefrontal cortex, amygdala and hippocampus, than in the controls. Further analysis showed stronger amygdala-hippocampus functional connectivity in the trauma-exposed healthy compared to the controls. Our findings revealed that survival of traumatic experiences, without developing PTSD, was associated with greater gray matter density in the prefrontal-limbic systems related to emotional regulation.

## Introduction

Trauma exposure is common and could increase lifetime vulnerability to mental health problems when individuals encounter stress or adversity, especially the most traumatic events, such as a massive earthquake, terrorism or war [1–3]. Although only a minority of humans develop posttraumatic stress disorders (PTSD) [4, 5], traumatic experiences predict an increased risk for psychopathology later in life in the general population [6–9]. Although previous studies have indicated that trauma exposure could impact brain function and structure in nonclinical individuals [10–12], few studies have focused on the long-term effects of trauma exposure in a nonclinical sample. Moreover, it is not yet clear how the long-term effects of trauma exposure cause the change of brain structure and function associated with emotion and memory processing in trauma-exposed individuals. Thus, the changes in brain structure and functions might help us understand the neural mechanisms underlying both vulnerable and resilient individuals in this nonclinical sample.

Extensive neuroimaging studies of patients with mood disorders have shown significant alternations in brain structure and function [13–16], which have also been found in healthy adults after stressful life events [10, 17, 18]. Other researches have indicated that the amygdala, hippocampus and ventral medial prefrontal cortex (vmPFC) play an important role in emotion regulation, memory and coping with stress responsively [19–24]. Animal studies also indicate that these brain regions are associated with emotion processing and regulation of the hypothalamic pituitary adrenal axis under stressful situations [25–28]. The amygdala is hyper-responsive in PTSD, which is associated with exaggerated fear responses and emotional arousal [29]. Exposure to stress could induce increased activity within the amygdala related to traumatic events [30, 31], and a hypo-responsive vmPFC and hyper-responsive amygdala are exhibited in anxiety-related disorders [32, 33]. Previous animal and human studies have shown that exposure to stress induces neural structural and functional abnormalities of the hippocampus associated with dysfunctional episodic and autobiographical memories [34–40]. Moreover, recent studies with nonclinical samples have also found decreased volume in the frontal-limbic regions, such as the hippocampus, anterior cingulate and medial prefrontal cortex, which were related to serious chronic life stress [41], closer proximity to the disaster on 9/11 [10] and more cumulative adverse events [17]. Additionally, some studies have indicated that the experience of acute events has a short-term effect on structure and function [11, 42] in the prefrontal-limbic, parietal and striatal brain systems. As a consequence, it is important to explore the long-term effects of experiences of acute events on brain structure and function and to better understand the neural circuits underlying resilience in these trauma-exposed individuals with no psychiatric disorders.

Rather than mass univariate analyses, multivariate-pattern-analysis (MVPA) is sensitive to spatially distributed effects and can be used to separate patients from the healthy controls using structural MRI (magnetic resonance imaging) data or the functional MRI data [43, 44]. Previous mass univariate analyses studies have reported differences between patients and controls at the group level. Unlike univariate analyses, MVPA neuroimaging studies generally trend to make inferences at the level of the individual rather than the group [45]. Apparently, mass-univariate and multivariate methods are complementary approaches. Thus, we combined the two types of analyses to reveal the neuroanatomical correlates of trauma-exposed individuals with no psychiatric disorders.

In the present study, more than 3 years after the 5/12 Wenchuan earthquakes, we used voxel-based morphometry (VBM) and resting state fMRI (rs-fMRI) to investigate the changes in brain structure and functional connectivity between the stress-related brain areas in trauma survivors compared to healthy controls.

## Methods

### Subjects

A total of forty-two healthy undergraduate students participated in this study about forty-one months after the 5/12 Wenchuan earthquake. Twenty-one individuals (14 female, ages 20.5 ± 1.3) from the Wenchuan earthquake disaster area were identified by the post-traumatic stress disorder self-rating scale (PTSD-SS) (with score < 60) as resilient trauma survivors [46]. The PTSD-SS was constructed based on the definition and diagnostic criteria of PTSD described in the Diagnostic and Statistical Manual of Mental Disorders: Fourth Edition (DSM-IV). And participants who have got the total score below 60 in PTSD-SS are thought of no serious PTSD symptoms [47]. PTSD-SS serve as a screening tool for PTSD. Subjects were excluded if they (1) had clinically significant PTSD symptoms (PTSD-SS total score over 60); (2) had undergone any form of psychotherapy or taken psychotropic medications after the Wenchuan earthquake; (3) or had only experienced psychotic illness through the files of mental health education. Moreover, twenty-one healthy controls (12 female, ages 21 ± 1.1) who were not exposed to the earthquake were recruited from the local campus by advertisements. The two groups were well matched for age (*p* = 0.24), sex (*p* = 0.58) and length of education (*p* = 0.62) (two sample t-test using SPSS, Inc., Chicago, IL, USA). Two participants were tested but excluded from the VBM analysis due to problems in the image registration.

The Impact of Event Scale Revised (IES-R) is a self-administered, 22-item questionnaire as indicators of PTSD. It should be administered due to trauma and have no other medical basis, and perhaps it is the most widely used self-administered assessment in the field of traumatic stress. The IES-R was administered after scanning for all subjects to record their current subjective distress. They were told: “Please read each item, and then indicate how distressing each difficulty has been for you during the past 7 days with respect to wenchuan earthquake [48].” The IES-R is not a diagnostic or screening tool for PTSD. We aimed to observe the degree of distress especially in resilient trauma survivors that they respond to the traumatic event. There are no specific cut-off scores for the IES-R and the higher scores are representative of greater distress.

The Spielberger State-Trait Anxiety Inventory (STAI) [49] was used to measure the difference in the trait component and current state of stress response between the two groups, a long time after the earthquake. All subjects had normal or corrected-to-normal vision and none had a history of neurological or psychiatric disease. The Southwest University Brain Imaging Center Institutional Review Board approved this study. Written informed consent was obtained from all subjects.

### Functional magnetic resonance image acquisition

All MRI data were performed on a 3T scanner (Siemens Trio, Erlangen, Germany) at the Brain Imaging Research Central at Southwest University. First, resting-state functional MR images were obtained using an Echo Planar Imaging (EPI) sequence with the following parameters: time repetition [TR] = 2000 ms; time echo [TE] = 40 ms; flip angle [FA] = 90, slices = 28, matrix = 64×64; field of view [FOV] = 192 mm; acquisition voxel size = 3.4 × 3.4 × 4 mm. A total of 242 volumes were collected for each subject. During fMRI scanning, subjects were instructed to close their eyes, not to move, think about anything particular, or fall asleep [50, 51]). Each subject reported not having fallen asleep using a simple questionnaire after the scan. Second, a high-resolution 3D Magnetization Prepared Rapid Gradient Echo structural image was acquired with the following parameters (TR/TE/FA = 1900 ms/2.2 ms/9°, resolution = 256×256 matrix, slices = 176, thickness = 1.0 mm).

### Functional magnetic resonance image processing

The resting-state data preprocessing was performed using a Matlab (Math works Inc., Natick, MA) toolbox Data Processing Assistant for Resting-State fMRI (DPARSF, http://resting-fmri.sourceforge.net/). This included the following steps: the first 10 functional images were discarded due to signal instability and the subject’s adaptation to the scanning noise. The slices of the remaining 232 volumes for each subject were corrected for different collection times of signals by slice time correction. Subsequently, the functional image time series were motion-corrected by realigning all images to the middle image volume. The individual structural image of each subject was co-registered to the mean functional image generated after motion correction. Third, the functional images were spatially normalized into the Montreal Neurological Institute (MNI) space using the transformation information generated by segmentation and resampled into 3 mm cubic voxels. The normalized images were smoothed with an isotropic Gaussian kernel (FWHM = 4 mm).

### Structural data preprocessing

The structural MRI images were processed using SPM8 software (Welcome Department of Cognitive Neurology, London, UK; www.fil.ion.ucl.ac/spm). For better image registration, the reorientation of the images was manually set to the anterior commissure. The structural image was then segmented into gray matter (GM), white matter (WM), and cerebrospinal fluid (CF). Subsequently, we performed diffeomorphic anatomical registration through exponentiated lie (DARTEL) algebra in SPM8 for registration, normalization and modulation. Then, the registered images were transformed to Montreal Neurological Institute (MNI) space. Finally, the normalized, non-modulated images (gray matter and white matter density images) were smoothed with a 10-mm full-width at half-maximum Gaussian kernel to increase the signal to noise ratio.

### Traditional univariate VBM analysis

The general linear model (GLM) approach was used to test the gray matter density (GMD) differences between the trauma-exposed group and the healthy controls. To control for the effects of age, gender, and whole brain gray matter density, these variables were added as additional covariates. We also applied absolute threshold masking (all voxels with GM values of < 0.2 were excluded) to avoid edge effects between gray and white matter. To explore whether there ware structural differences in the amygdala, parahippocampal and dACC, which have been considered to be involved in emotion regulation and stress responsively [19–24], small volume corrections (SVC) were applied using masks created using the WFU PickAtlas toobox [52–54]. Finally, to further investigate the predictive ability of regional gray matter density, the significant differences map (Height threshold: p < 0.005; Extent cluster threshold: p < 0.05, with a whole brain FWE-corrected) and separate clusters obtained from the univariate analysis were used as a region of interest mask, and we used Support Vector Machine (SVM) [55] to discriminative between trauma-exposed group and healthy controls.

### Multivariate pattern analysis based on VBM

The PRONTO toolbox based on pattern classification techniques was used for the analysis of neuroimaging data [56, 57]. The dataset is usually divided into two sets: training and testing. During the training phase, an algorithm learns some mapping between patterns and the labels [56]; during the test phase, the learned function is used to predict the group membership of the test individuals. To make the test data set independent from the training set, a mean centering and leave one out cross-validation (LOOCV) procedure [58] was performed for each subject. Because the variance of the importance weights can be large or even infinite, leave-one-out may lead to an unreliable estimate [59–61]. However, it provides an almost unbiased estimate of the generalization ability of a classifier [61]. Most of statistical theory and machine learning theory are based on the assumption that the data is independently and identically distributed. However, in neuroimaging research, this assumption is often not met [62]. Therefore, classical estimates of confidence intervals may not always be appropriate. Permutation testing is a non-parametric procedure that allows to obtain meaningful confidence intervals and p-values in this case [62]. In order to obtain more meaningful confidence intervals and p-values of each cluster, a random permutation test (10,000 times) was used to examine the statistical significance of the classification models [63, 64].

### Functional connectivity: Region of interest selection and data analysis

Functional connectivity was performed by applying a seed region correlation approach [65] using the Resting-State fMRI Data Analysis Toolkit (REST) software package [66]. Previous studies have shown that acute stress was closely interrelated with structural or functional alternation of the amygdale, medial prefrontal cortex and hippocampus [19–24]. Therefore, both sides of the three brain areas were chosen as the regions of interest (ROI) using a previously validated, anatomically labeled (AAL) template image [53]. Subsequently, the band pass filtering (0.01–0.08 Hz) and linear detrending were performed. The time courses for the various covariates (white matter, cerebrospinal fluid, and 24 motion parameters for head movement) were extracted and regressed out to cancel out the potential impact of physiological artifacts. Here, the Friston 24-parameter model, which includes 6 current head motion parameters, 6 head motion parameters from the previous imaging volume, and the 12 corresponding squared items, was utilized to regress out head motion effects from the realigned data based on previous reports demonstrating benefits of higher-order models in reducing head micromovements [67, 68]. Then, we implemented a “scrubbing” procedure to censor high motion volumes [69, 70]. The time course for each ROI was extracted by averaging the time series of all voxels within each ROI. Finally, the correlation coefficients were transformed to z-values using Fisher's r-to-z transformation to improve the normality of the partial correlation coefficients [71, 72]and to enable group comparisons. In the group comparison analysis, we tested for strength connection (*z*-transformed *r* value) between the trauma survivors and the healthy controls. To investigated whether the functional connectivity (amygdala-medial prefrontal cortex, amygdala-hippocampus and medial prefrontal cortex-hippocampus) would be affected by the IES-R scores between the earthquake exposed group and the control group, we calculated the Pearson correlation coefficients between the z-scores of each ROI-to-ROI and the IES-R scores with controlling the sex and age as regressors of no interest.

## Results

### 5/12 earthquake-exposed versus comparison group

The levels of anxiety were within the normal range across all groups, and the STAI scores were not significantly different between the trauma survivors and healthy controls (Table 1). Age, sex and length of education were not significantly different between the two groups. The PTSD-SS scores of the trauma survivors were significantly higher than the healthy controls’ (t = 4.3; p< 0.001). The IES-R scores for the healthy control group fell well within the normal range, and were significantly lower than trauma survivors’ (t = -3.5; p< 0.001).

**Table 1:** Demographic Characteristics of trauma Survivors and healthy controls.

Characteristics	Survivors ± SD (n = 21)	Controls ± SD (n = 21)	*P*
Female to male, no	14:7	12:9	p = 0.58
Mean age, y	20.5 ± 1.3	20.9 ± 1.1	p = 0.24
Years of education	13.7 ± 1.4	13.9 ± 1.1	p = 0.62
PTSD-SS Scores	46.5 ± 11.3	32.9 ± 7.7	p < 0.001**
STAI(S-AI) Scores	39.7 ± 6.8	43.7 ± 9.9	p = 0.14
STAI(T-AI) Scores	41.7 ± 7.5	46 ± 11.2	p = 0.15
IES-R Scores	24.3 ± 15.1	11.5±5.8	p < 0.001**

### The VBM results of univariate analysis and MVPA

**Fig 1:**
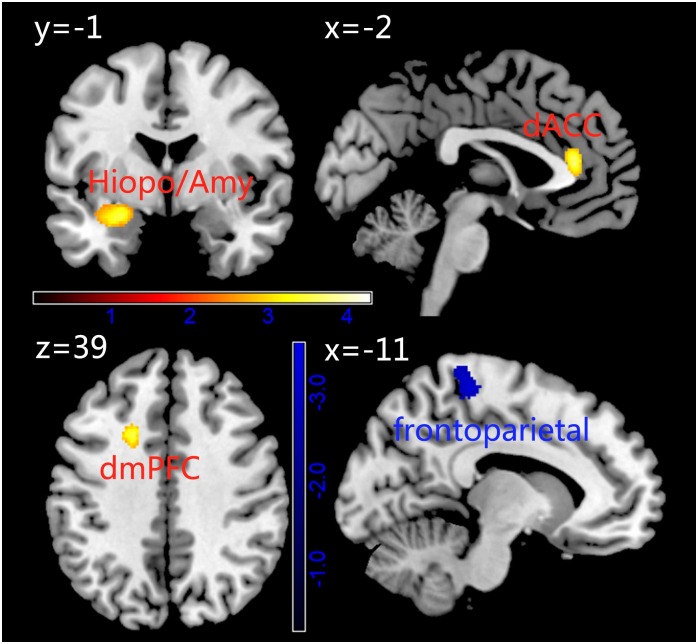
Differences in gray matter density (GMD) between Trauma Survivors and Healthy Controls using univariate analysis based on voxel-based morphometry. The hot in the map represent represents the results of GMD Trauma > Control. While, the blue represents the result Control > Trauma.

Compared with the healthy controls, the trauma survivors showed that greater GMD in the dorsal medial frontal cortex (dmPFC), dorsal anterior cingulate cortex (dACC) extended to the rostral anterior cingulated cortex (rACC) and bilaterally in the hippocampus/amygdala and lower GMD of the frontoparietal association cortex (Fig 1 and Table 2).

**Table 2:** The differences in gray matter density between trauma survivors and controls.

Brain structure	cluster	t	MNI		
			x	y	z
Trauma survivors > Controls					
L dMPFC*	528	3.69	-20	14	38
dACC***	1005	4.29	19	26	17
Parahippocampal/Amygdala**	498	3.56	-27	-3	-15
Trauma survivors < Controls					
Frontoparietal cortex *	612	3.88	-23	-38	75

* Height threshold p< 0.005, uncorrected.

** p < 0.01, small volume corrected.

*** p < 0.005, small volume corrected.

**Fig 2:**
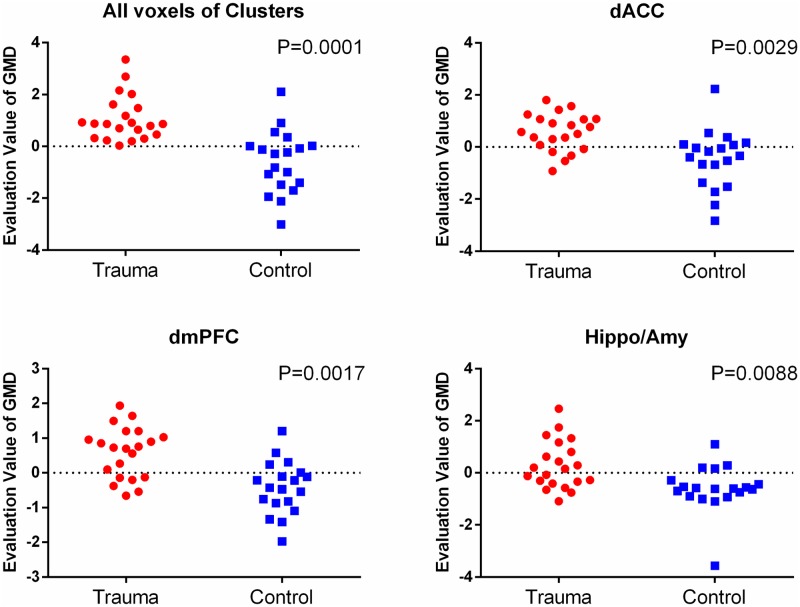
The classfication plot of individual diagnosis results. A support vector machine was used to construct multivariate models and to classify participants as trauma survivors or controls. Evaluation value of GMD: the output value of the machine’s decision function for each test sample. The decision threshold is displayed by a horizontal line at the centre of the plot. Statistical significance P-values were generated by using permutation test (n = 10,000).

We also used MVPA to further investigate the predictive ability of regional gray matter density at the individual level, based on the GMD differences map (GMD map) and regions of interest obtained from the univariate analysis. The applications of SVM to dmPFC clusters classified the trauma-exposed and healthy control groups with a sensitivity of 71.4% and 73.7%, respectively, leading to an overall accuracy of 72.5%; dACC (76.2%, 68.4%, 72.5%); hippocampus/amygdala (52.4%, 78.9%, 65%). When all these clusters were considered simultaneously, it classified the trauma-exposed and healthy control groups with a sensitivity of 100% and 68.4%, respectively, leading to an overall accuracy of 85%. Permutation testing indicated that these predictive accuracies were all statistically significant (see Fig 2).

### The Strength of connections between ROIs and impact of event scale revised

**Fig 3:**
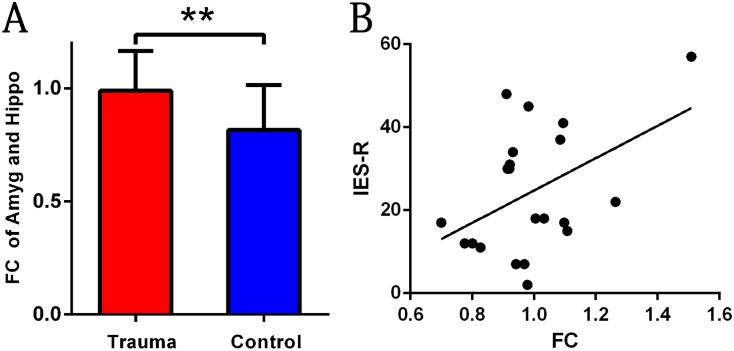
Differences in Functional Connectivity Maps for Trauma Survivors compared with Healthy Controls. (A) Trauma survivors showed significant stronger functional connectivity of the amygdala-hippocampus. (B) The correlation between the strength of the connection and IES-R scores in the trauma survivors.

A group comparison analysis revealed that there were no significant differences in the functional connection of the amygdala with the mPFC between the two groups (t = -1.56; p = 0.126). However, an increased strength of the connection of the amygdala with the hippocampus was found in the trauma survivors compared to the healthy controls (*t* = 2.86; *p* = 0.007, Fig 3A). Additionally, there was a significant positive correlation between the strength of the connection and IES-R scores in the trauma survivors (*r* = 0.45; *p* = 0.04, Fig 3B).

## Discussion

In the current study, we investigated the long-term effect of the traumatic event (the Wenchuan Earthquake) on human brain structure and functional connectivity. We found that resilient trauma-exposed survivors showed greater gray matter density in prefrontal-limbic brain systems and lower gray matter density in the frontoparietal association cortex than controls. Resting-state functional connectivtiy analysis found that resilient trauma-exposed survivors showed strengthened functional connectivity between the amygdala and hippocampus compared to controls.

The prefrontal cortex plays an important role in top-down emotion regulation and it can regulate the neural activity of the limbic systems, especially the amygdala and hippocampus [20, 21, 73–75]. The previous review which focused on the neuroimaging findings in PTSD patients has suggested that volume reductions in the prefrontal cortex might be related to a reduced capacity to inhibit fear and modulate affective responses [76]. Similar studies have revealed that resilient trauma survivors showed an increased gray matter volume in the right middle prefrontal gyrus compared with PTSD patients [77] and that healthy subjects who experienced the traumatic event showed increased resting-state activity in the left lateral prefrontal cortex compared to healthy controls [11]. Moreover, the structural abnormalities in the dACC and amygdala may reflect predisposed neural abnormalities that increased the likelihood of developing PTSD following exposure to trauma [78]. Sekiguchi et al. revealed that subjects with lower gray matter volume in the ACC before the earthquake were likely to have PTSD symptoms [79]. Thus, combine our results, greater gray matter density in the prefrontal-limbic systems might be associated with the better ability to control the sustained hyper-activation in limbic systems due to the traumatic experience [42].

It has recently been highlighted that periods of traumatic stress might alter the structural and activity in the human parietal lobule [77, 80–83]. An MRI study found that resilient trauma survivors showed less gray matter volume in the parietal cortex compared to healthy controls [81]. Interestingly, there was a pattern of increases cerebral blood flow in PTSD patients and decreases in resilient trauma survivors in the parietal cortex with exposure to traumatic pictures and sounds [82]. These findings suggested that the frontoparietal association cortex is sensitive to traumatic stress. The frontoparietal association cortex was implicated in the visual imagery representing, an important component of the visuospatial processing of preparation for responding to a physical threat, as well as a critical component of flashbacks and similar PTSD symptoms. Therefore, combine our results with previous studies, the density reduction in the frontoparietal association cortex might be related to reduced flashbacks and non-excessive reaction to a threat.

Furthermore, trauma not only induces an anxious state and emotional arousal but can also impair memory through the amygdala’s interactions with other brain regions [27, 84, 85]. The hippocampus is widely implicated in memory encoding and maintenance, forming and storing memories associated with emotional events [86, 87] and autobiographical memory [88, 89]. Many studies have shown abnormal hippocampus activity [19, 90] and decreased hippocampal volume [37, 91] in stress-related pathologies, such as major depressive disorder and PTSD. Resilient early life stress subjects yet showed increased degree of the hippocampus in graph network relative to healthy controls [92]. In addition, decreased hippocampal volume is also associated with serious and long-lasting traumatic stress [93]. In this study, we have observed increased functional connectivity between the amygdala and hippocampus in the resilient trauma survivors. Previous studies suggested that increased amygdala-hippocampus correlations during recollection of negative autobiographical memories [94, 95] or emotional events [94, 96]. For example, Smith et al. (2004) showed that retrieval of emotionally valenced contextual information is associated with increased connectivity between the hippocampus and amygdala [97]. Greenberg et al. observed co-activation of the amygdala and hippocampus during autobiographical memory retrieval task (recollection of episodes from the personal past) [98]. This hippocampal-amygdala effects in autobiographical memory also were found in other studies [99, 100]. Admon et al. (2009) showed that amygdala activity could predict the subsequent reactivity of the hippocampus in resilient trauma-exposed individuals [38]. Several studies indicated that the connectivity between the amygdala and hippocampus is associated with the modulation of stress effects on memory consolidation, memory retrieval [24, 101–103]. Thus, increased connectivity between the amygdala and hippocampus may store and maintain negative autobiographical memory. Moreover, resilient trauma survivors can retrieve autobiographical memories more specifically than PTSD patients [104]. The connectivity strengths might imply one potential mechanism by which increased functional connectivity between the amygdala and hippocampus indexes lower risk for PTSD.

However, we observed a significant positive correlation between the connectivity strength and distress. It is not consistent with the notion that greater connectivity serves as a protective role in developing PTSD. Thus, this correlation should generally be interpreted with caution. To begin with, the range of the symptom severity might not large enough to explore the brain-behavioral relationship. Perhaps there would be an inverse association between symptoms and hippocampus-amygdala connectivity in subjects with high-level symptoms. Besides, the trauma-exposed condition is characterized by other three typical symptom clusters: re-experiencing, avoidance and hyperarousal symptoms [105, 106]. It is possible to observe the significant negative correlations between other specific symptom clusters and the connectivity strength. Last but not least, the correlation between distress and connectivity might not be explained by linear regression. Perhaps, performing the nonlinear correlation analysis would be more acceptable. These interpretations remain largely speculative and need further investigation.

Our study revealed that resilient trauma survivors showed greater gray matter density in the prefrontal-limbic systems that were implicated in emotional regulation. The emotional regulation ability plays a critical role in preventing the onset of PTSD in those trauma-exposed nonclinical adults. However, there are two possible explanations for the current findings. One possibility is that these structural differences might be a pre-existing factor and those participants did not develop PTSD due to these biological protective factor. Nevertheless, we cannot rule out the possibility that the structural differences are the brain “scar” after the traumatic event. Our study is quite preliminary in nature and only longitudinal studies that examine the changes in behaviors and neuroimaging measures in individuals before and after the traumatic stress could fully rule out one of the possibilities.

Of note, there are other limitations in our study. First at all, we did not include the participants who experienced the Wenchuan earthquake and then developed the PTSD in our study. This limitation restricted our ability to differentiate the resilience and vulnerability factor at the neural level. We expect to recruit an additional PTSD patients group and carry out the longitudinal project in the future. Besides, the sample size of our study was modest and the range of symptoms severity is limited. This point largely affects the reliability of our brain-behavior correlational analysis. Finally, the assessments of symptom in this study are not comprehensive enough. The assessments should be multiplex and include different symptoms clusters, such as re-experiencing, avoidance and hyperarousal symptoms along with a high rate of dissociative symptoms [107, 108].

Our results are largely consistent with findings from studies on other stressors (and stress in general) in the literature [11, 19–24, 77]. Although the sample size of the study was modest, we have provided a reliable and valid stress model especially for gray matter density effects through MVPA analysis. Previous studies mainly focused on the short-term effect of trauma exposure or patients with PTSD, while our study investigated the long-term effects of trauma exposure in a nonclinical sample. Furthermore, our findings revealed the structural and functional differences in brain regions that are usually implicated in emotional regulation. In conclusion, our study revealed that survival of traumatic experiences, without developing PTSD, was associated with greater gray matter density in the prefrontal-limbic systems related to emotional regulation.

## Supporting Information

S1 File Behavior data of trauma Survivors and healthy controls. (XLSX)
